# Removal of a Horizontally Displaced Dental Implant below the Mandibular Canal

**DOI:** 10.1155/2023/6663874

**Published:** 2023-03-24

**Authors:** Khodor Ahmed Fakih, Maria Stiliyanova Edreva, Biser Borisov Stoichkov

**Affiliations:** ^1^Department of Oral and Maxillofacial Surgery, Faculty of Dental Medicine, Medical University, Sofia, Bulgaria; ^2^Denta Puls Clinic, Sofia, Bulgaria

## Abstract

A clinical case of a horizontally displaced dental implant, which moved below the level of the mandibular canal during surgery is presented together with a brief review of the comparable published cases. The bone mineral density and the morphology of the alveolar ridge were analyzed at the site of osteotomy, and the low bone density of 265.32 ± 86.41 Hounsfield Units was found in the area. The factors related to implant displacement were: the anatomical features of bone structure, and the applied mechanical pressure during the implant insertion. The displacement of the dental implant below the level of the mandibular canal during implantation can be a severe complication. Its removal requires the safest surgical approach to avoid damaging the inferior alveolar nerve. The description of one clinical case does not provide grounds for drawing definite conclusions. To avoid similar incidents, detailed radiographic assessment before implantation is necessary; it is also important to follow the surgical protocols of implant placement into soft bone and to create conditions for a good visibility and sufficient control of bleeding during surgery.

## 1. Introduction

Over the last decade, implant dentistry has become an integral part of dental medicine and helped clinicians to improve the quality of life for a large group of patients [1, 2]. Although implant-supported restorations can often be a convenient alternative to existing therapeutic options, in some cases, they are the first method of choice for the rehabilitation of severe functional, anatomical, or aesthetic problems resulting from the loss of natural teeth [3]. Dental implant placement in the distal edentulous parts of the mandible is a routine, usually safe, and predictable procedure with sufficient surgical competence and favorable anatomical preconditions [4, 5]. It is necessary to have a sufficient bone volume in the area of implantation both in the vertical and horizontal directions. Compared with other parts of the jaws, the largest bone volume deficiency can often be observed in the posterior areas of the mandible due to the early loss of the lower molars and the onset of alveolar bone atrophy [6]. In most cases, posterior areas are characterized by large morphological variations [7, 8]. Usually a thick layer of compact bone is embracing the trabecular bone [9]. The average volume of trabecular bone in this area varies from 20.9 to 36.9% [10]. With advancement of bone atrophy, both the quantitative and qualitative characteristics of the alveolar bone can be changed [11, 12]. In the analysis of biological apatites and crystallites in the distal edentulous areas of the mandible, Furukawa et al. [13] indicated that the tooth loss and degree of alveolar bone resorption affect the quality of the bone in these places. The mandibular bone resorption depends to a greater extent to the presence or absence of teeth than the patient's sex and age.

The structural characteristics of the bone, such as mineral density and maturity, are important for its strength. They provide primary stability of the implant while achieving maximum bone-to-implant contact (BIC) and is important for successful osseointegration [14, 15], as well as dental implant treatment planning [16, 17]. Unfortunately, in a clinical practice all specific risk factors that may develop during the surgical stage are sometimes overlooked, such as local conditions that have an unfavorable effect during the surgical stage increasing the risk of complications or unsuccessful implantation.

Displacement of a dental implant into the body of the mandible is a severe but rare complication [18] compared with other well-known complications during the surgical stage such as: hemorrhage, damage to the lower alveolar nerve, damage to the adjacent tooth, lack of primary stability, and implant dislocation in adjacent soft tissue spaces [5, 19–21].

Following the PubMed search, 18 cases of dental implant displacements into the body of the mandible were found and described in eight articles. In the literature review of Kim et al. [2], the implant dimensions, direction of its displacement, patient's sex, and presence of osteoporosis were traced in 15 cases. The majority of those cases (14 cases) included women, 3 of them had a diagnosis of osteoporosis. As a complication, hypoesthesia with varying severity of the alveolar nerve was reported in seven cases after removal of the implants. The directions of implant displacement were same or similar to the axis of the original osteotomy; in eight cases the implants were displaced in the lingual direction relative to the lower alveolar nerve [2, 4, 6, 22], in two cases—buccally [2, 19], in three cases—vertically [23, 24], and in the other five cases the direction was not described. The X-ray examinations before implant placement revealed no bone defects, reduced bone density, or other pathology at the sites of implantation.

The dental implant displacement into the body of the mandible is closely related with the bone morphology at the site of implantation. The bone mineral density and primary stability of dental implants are closely related [25, 26]. The blood supply is smaller in the D1 type dense bone and the surgical trauma is higher during osteotomy preparation, which worsens conditions for osseointegration despite usually achieved higher primary stability [27]. However, a higher rate of local complications was observed in softer bone types due to low primary stability [11]. This affects selection of the surgical protocol and duration of osseointegration [28]. The cone beam computed tomography (CBCT) sometimes does not provide direct correlation between the gray values and the Hounsfield Units (HU), which describe bone mineral density.

In most cases, the edentulous distal parts of the mandible are characterized by high mineralization [25, 28, 29]. Low bone density of trabecular bone is observed only in 15–20% of the cases [27, 30]. Accidental implant displacement in the posterior mandible is mostly associated with overpreparation of the implant site, subcrestal implant positioning, poor BIC, and insufficient or a lack of primary stability [2, 18, 23, 24]. The low bone quality and density contribute to implant displacement during surgery [6].

## 2. Case Presentation

A 36-year-old patient accompanied with his dentist visited the Denta Puls Clinic (Sofia, Bulgaria) on January 17, 2020 for an emergency. According to their anamnestic data, an attempt was made to place two implants (ICX-premium, Medentis Medical GmbH, Bad Neuenahr-Ahrweiler, Germany, 4.1 mm × 8.0 mm) in the distal area of the right mandible (teeth #46 and 47). A two-stage surgical protocol for implantation was performed using a mucoperiosteal flap elevation. The bleeding had been extensive. Two osteotomies were performed in the lower right molars. After the machine-driven insertion of the first implant in the first lower right molar (tooth #46), the bleeding continued. During the placement of the second implant, using machine-driven insertion, the implant “sinked and disappeared in the bone” in the area of the second lower right molar (tooth #47), causing an increase of bleeding. Therefore, the surgical procedure was terminated, and the patient was brought to the clinic.

**Figure 1:**
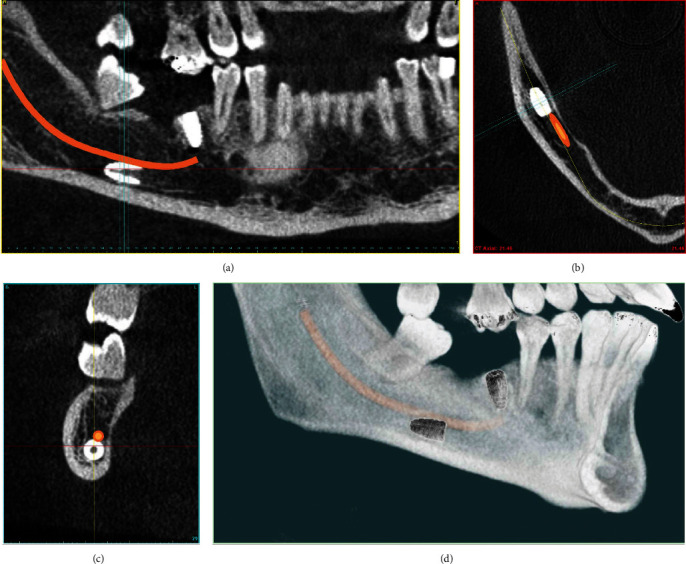
CBCT after implant dislocation. (a) Reformatted panoramic image. (b) Axial reconstruction. (c) Sagittal reconstruction. (d) 3D reconstruction of the mandible (lingual view). All images show that the implant is located below the level of the mandibular canal.

During the clinical examination (1) it was found that there was a freely un-adapted mucoperiosteal flap; (2) profuse bleeding in the area of placement of the second implant and in adjacent soft and bone tissues; and (3) an uncovered bone at the site of surgery. The CBCT examination revealed the presence of one vertically positioned implant in the area of tooth #46 and one horizontally displaced implant below the level of the mandibular canal (Figure 1).

The bleeding was stopped urgently without removing the displaced implant under local anesthesia [inferior alveolar nerve (IAN) block]. Lavage and debridement of the wound were made and at and around the site of the displaced implant, an absorbable oxidized cellulose gauze (Gelita-Cel Standard, Gelita Medical GmbH, Eberbach, Germany) moisturized with Etamsylate, 250 mg/2 mL (Dicynone, Sanofi Winthrop Industrie, Paris, France) was used as local hemostatic. The flap was adapted and sutured tightly. Additionally, two ampules of Etamsylate, 250 mg/2 mL were administered intramuscularly to control and prevent possible postoperative bleeding. The hemorrhage was stopped completely. Amoxicillin (875 mg) and clavulanic acid (125 mg) were administered twice daily, along with (calcium carbonate, vitamin K2, and vitamin D3) 2 × 1 tablets of Kalcikinone for 10 days, as antibacterial and hemostatic therapy. A blood test was conducted immediately after surgery. The indicators related to the hematological status of the patient were examined. Based on their reference values, no abnormalities were found. This was also confirmed following consultation with a hematologist. The case was monitored under outpatient conditions till the 10th day when the sutures were removed. There were no complications during the postoperative period. The patient had no sensory disorders. The surgical procedure for removal of displaced implant was planned at the end of February 2020. The patient lived abroad and there were strict epidemic restrictions (including travel and elective surgery) related to COVID-19 pandemic; thus, the surgery was postponed until the epidemic measures were relieved. These restrictions were partially removed in July 2020. After 7 months, the surgery was scheduled to remove the implant, and the patient provided the relevant consent. The operations goals were as follows:

1. To determine the condition and stability of the implant in the first lower right molar (tooth #46).

2. Removal of the displaced dental implant in the second lower right molar (tooth #47).

3. To prevent damage to the neurovascular bundle of the mandible during surgery.

**Figure 2:**
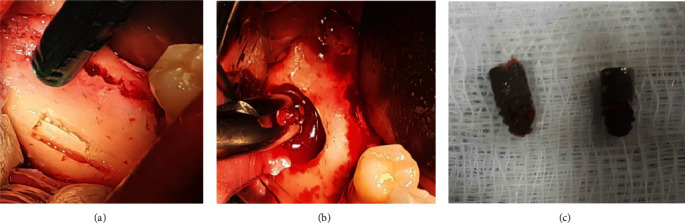
(a and b) Stages of operation to remove the dislocated implant. (c) The removed implants.

The CBCT was performed again and it was found that the displaced implant remained in its position and there were no signs of osseointegration. In August 2020, the surgery was performed using an IAN block on the right side of the mandible (articaine with epinephrine 1 : 100,000, Septodont, Saint-Maur-des-Fossés, France). A full-thickness mucoperiosteal flap was elevated from tooth #45 to tooth #48. Significant bone resorption was found around the implant in tooth #46, spanning approximately one-third of its length; implant mobility was also observed. It was decided to remove the implant. Using an ultrasonic surgical device (Piezosurgery touch, Mectron S.p.A., Loreto, Italy), a horizontal cortical bone window was formed using OT6 and OT7 tips (Mectron S.p.A.), in the lower part of the body of the mandible, parallel to the dislocated implant (Figure 2(a)). The implant was identified, carefully released from the surrounding soft trabecular bone, and removed without damaging the integrity of the mandibular canal (Figures 2(b) and 2(c)).

**Figure 3:**
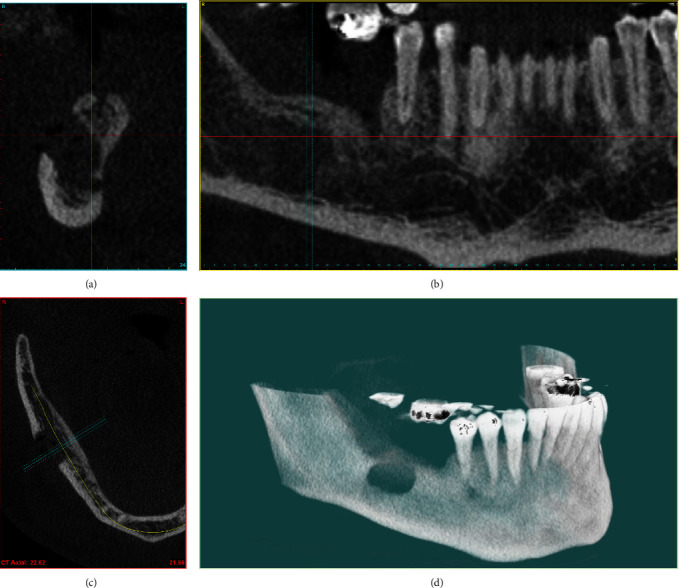
CBCT after implant removal. (a) Sagittal reconstruction. (b) Reformatted panoramic image. (c) Axial image. (d) 3D reconstruction.

The bone cavity was irrigated with saline solution, and swabs of absorbable oxidized cellulose (Gelita-Cel Standard, Gelita Medical GmbH) were placed. The flap was adapted and sutured with Vicryl Sutures 4-0 (Ethicon, Johnson & Johnson Medical, Irvine, CA, USA). Antibiotic therapy with Clinadamycin (2 mg × 600 mg) was prescribed for 10 days. A postoperative CBCT examination (Figure 3) was performed and confirmed that the right posterior side of the mandible was without the implants. The wound healed without complications. The sutures were removed on the 10th postoperative day. The goals of the operation were successfully achieved. A new implant placement was recommended in the same area after 6 months.

**Figure 4:**
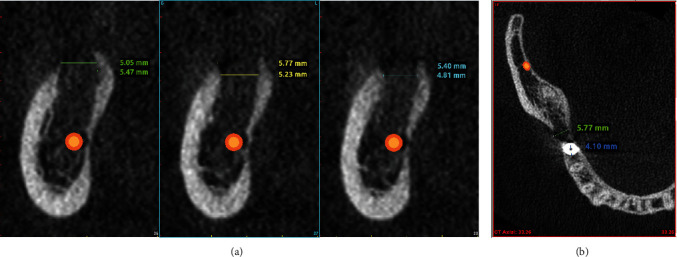
CBCT data after implant displacement. (a) (left) Vertical cuts. (b) (right) Axial reslice. Measurements of the implant bed at the site of displaced implant. The data show that the osteotomy dimensions are significantly larger than the diameter of the implant used. The lower border could not be determined.

To determine the possible local causes of this complication, we analyzed the available data from CBCT acquired immediately after the incident (January 17, 2020). We examined the boundaries of osteotomy at the site of the displaced implant. Overpreparation was detected at the implant site, both in vertical and horizontal dimensions, including the cortical bone plate at the top of alveolar ridge (Figure 4).

**Figure 5:**
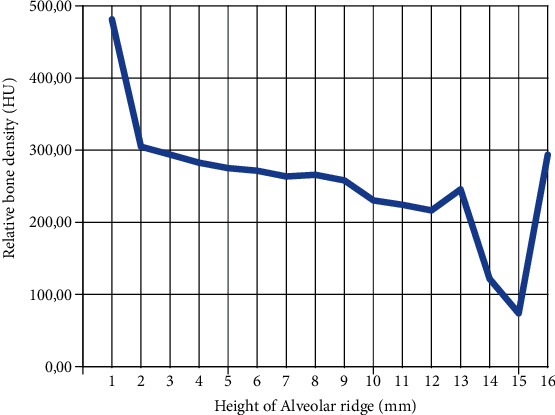
Distribution of relative bone density considering height of the alveolar ridge, next to the osteotomy area. The sharp decrease in bone density from the 13th to 15th mm corresponds to the position of the mandibular canal.

Additional analysis was made to determine the bone density along the periphery of osteotomy. The measurements of the bone density were made in the distally unaffected part, parallel to the osteotomy, with an area of 4.06 mm^2^. The measurements included all axial sections in the implantation area with a thickness of 1 mm: starting from the ridge of the alveolar crest to the lower border of the mandibular canal. The software was Simplant Pro 15 (Materialise, Leuven, Belgium). The statistical analysis of the data was made using the IBM SPSS Statistics 25 (10504-1722, Armonk, NY, USA). Extremely low bone density was detected in the observed area with an average value of 265.32 HU, standard deviation 86.411, and *n* = 16. The cross section results of the measurements are shown in Figure 5.

The data presented in the graphs above show that at the top of the alveolar ridge, the bone density has the highest detected value—481 HU; it sharply decreased after reaching 2 mm in depth. In the next sections (located at 3–12 mm), the values continue to decrease (from 293 to 216 HU). A slight increase in the bone density (245.16 HU) was found in the upper border of the mandibular canal—13 mm below the initial osteotomy site. Unfortunately, similar analysis could not be performed at the same location on the left side because of the presence of teeth.

## 3. Discussion

Kim et al. [2] reported that they found an almost-missing trabecular bone in the area of implantation in five cases. The low bone quality and low mineral density are the main causes of implant displacement during surgery [3, 4, 6, 18, 31]. In the present case, the average mineral bone density (265.32 ± 86.41 HU) was twice lower than the average reported values in this area—455–642 HU [1]; 556 ± 80 HU [26]; and 628.0 ± 20.19 HU [30]. Furthermore, in the distal parts of the mandible, even in the same area of implantation, the bone density along the osteotomy was different. The morphological analyses of such sections indicate that the highest bone density was found at the top of the alveolar ridge, which comprises a dense and compact bone layer. Below, the bone density decreased progressively with depth increase [9, 23] and increased slightly in the upper and lower borders of the mandibular canal [7]. Thus, it was necessary to avoid excessive pressure during implant placement.

In the cases of low bone density, it is possible to follow another surgical approach and perform osseodensification. These protocols include underpreparation of the implant bed and the usage of osteotomes for bone condensation or different specific instruments as bone expanders [32]. Thus, the final preparation of the osteotomy is achieved primarily by condensation [33]. This surgical technique is mainly applied to the distal parts of the maxilla. However, it can be assumed that there is a limit beyond which overcompression of the bone will impede osseointegration [34]. The bone compression of the implant bed shows different patterns of osseointegration depending on its extent. Excessive bone compaction should be avoided because it may impair implant osseointegration [35]. The primary stability achieved depends most on bone quality and quantity, osteotomy preparation, the implant surface, and its shape [34].

The factors associated with dislocation of the dental implant in the body of the mandible can be divided into the following groups: (1) anatomical features of the bone in the area of implantation, (2) mechanical pressure during implant insertion, and (3) precise planning of implant osteotomy technique and sufficient surgical competence and experience [5, 22, 23]. The available bone volume, local morphological features of the bone, and the number and type of implants determine the type of surgical approach [36]. According to Cardoso et al. [3], Oh et al. [6], and Doh et al. [23] the accidental displacements in the posterior mandible are associated with overpreparation of the implant site, low bone density, poor primary stability, insufficient planning, and an inadequate surgical technique. In addition, the described case also had inadequate bleeding control since the start of surgery. This worsened the visibility in the operative field and probably contributed to the over-preparation of the implant bed. The subcrestal implant positioning in the distal areas of the mandible poses an additional risk factor due to the possibility of losing contact between the implant and the hard lamellar plate of the alveolar bone. This may cause a decrease or sometimes loss of the implant's primary stability. Unlike in the other similar cases, the direction of implant displacement was unusual—it was displaced and it reached its final horizontal position under the IAN, which is probably due to the presence of an undiagnosed bone defect and low bone density. When there are no complications with a displaced implant and no infection, sometimes it be left in its displaced position in bone to osseointegrate. However, it was decided to remove both implants in the presented case, although there were no sensory complications. The implants were not fully osseointegrated and showed a certain amount of radiolucency. Due to a possible risk of inflammatory processes in the surrounding bone, and radiolucency diagnosed around the displaced implant it was decided to remove it.

Removing a displaced dental implant from the body of the mandible is a safe surgical procedure when done by a professional in dental surgery [2, 4, 19, 31]. Increased incidence of hypoesthesia is reported in cases of lingually displaced implants considering IAN [2]. Despite the unusual horizontal position in the described clinical case, the implant was not displaced lingually (Figure 1) from the IAN. No sensory disturbances were reported after surgical removal of the displaced implant using applied lateral approach [2, 4, 6, 22, 23]. The low location of the bone window did not reduce the available bone volume above the alveolar nerve [22]. Thus, new implant insertion and implant-supported restoration, which were scheduled afterwards are not expected to be complicated after surgery of removing displaced implant.

## 4. Conclusion

The displacement of the dental implant below the level of the mandibular canal during implantation can be a severe complication. Its removal requires the safest surgical approach to avoid injury of the IAN. The presented clinical case cannot be used to draw definitive conclusions. Multiple reasons can influence the implant displacement. Besides, the proven unfavorable bone morphology, low bone density, overpreparation of the implant site, and high pressure during implant insertion, and the impaired visibility due to inadequate bleeding control during the surgery also contributed. To avoid similar incidents, detailed CBCT assessment before implantation is necessary; the surgical protocols of implant placement into soft bone should be followed and good visibility and sufficient control of bleeding during surgical procedure should be obtained.

## Data Availability

The data of the bone density analysis, used to support the findings of this study are included within the supplementary information file. It is also available from the corresponding author on request at: biser.stoichkov@fdm.mu-sofia.bg.

## Supplementary Materials

Supplementary Materials A PDF file (*Additional_Information-from-the-bone_density-examination.pdf*) contains 22 CBCT axial images with measurements of the bone density in the distal adjacent area of the osteotomy and a table describing the exact positions and values of the traced indicators.
