# Supplementary material for: Removal of a Horizontally Displaced Dental Implant below the Mandibular Canal
Source: Case Rep Dent. 2023 Mar 24;2023:6663874. doi: 10.1155/2023/6663874 (PMC10065860; doi:10.1155/2023/6663874)
Supplement: Supplementary Materials — A PDF file (Additional_Information-from-the-bone_density-examination.pdf) contains 22 CBCT axial images with measurements of the bone density in the distal adjacent area of the osteotomy and a table describing the exact positions and values of the traced indicators. [file 6663874.f1.pdf]

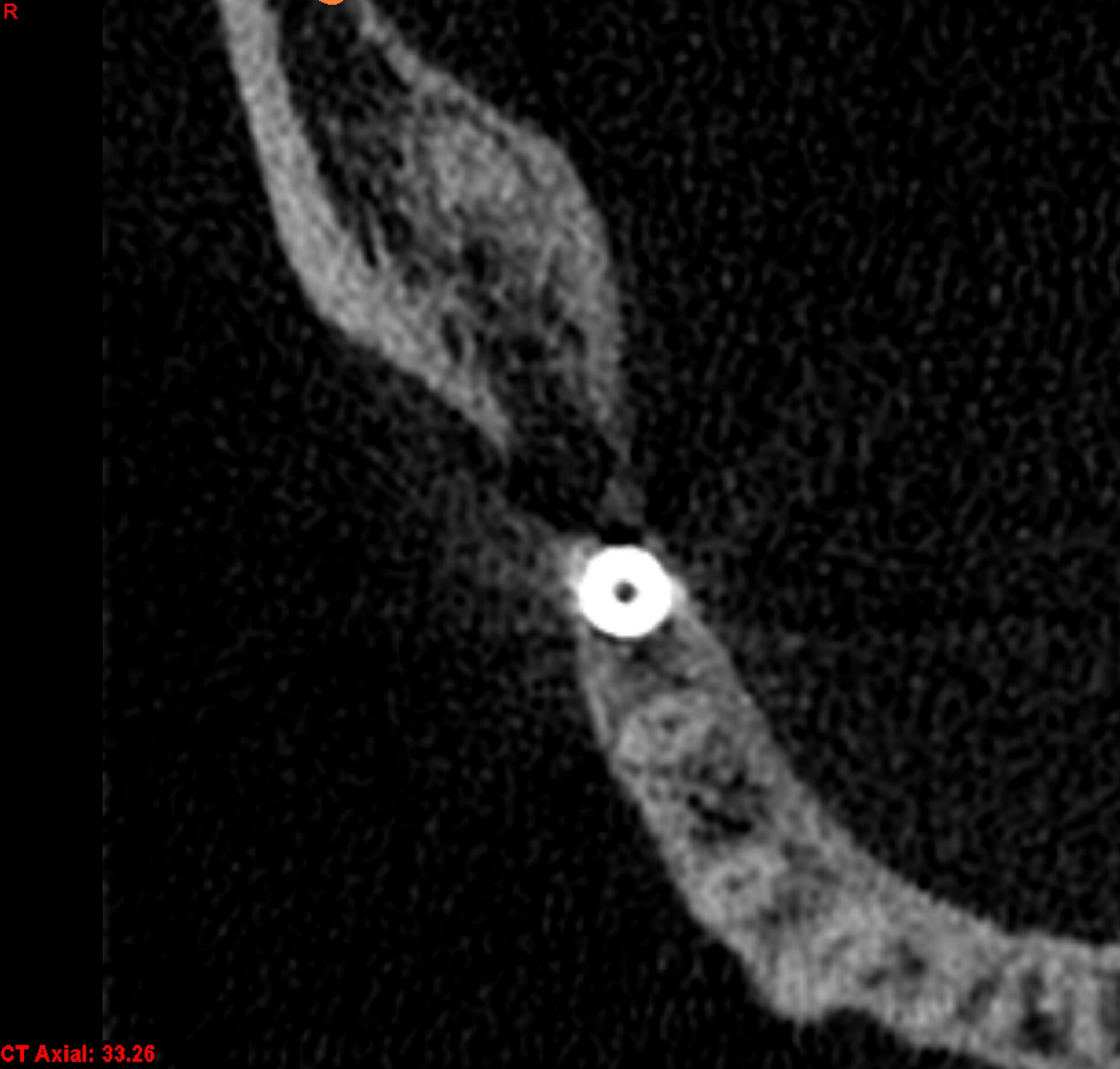
R
CT Axial: 33.26

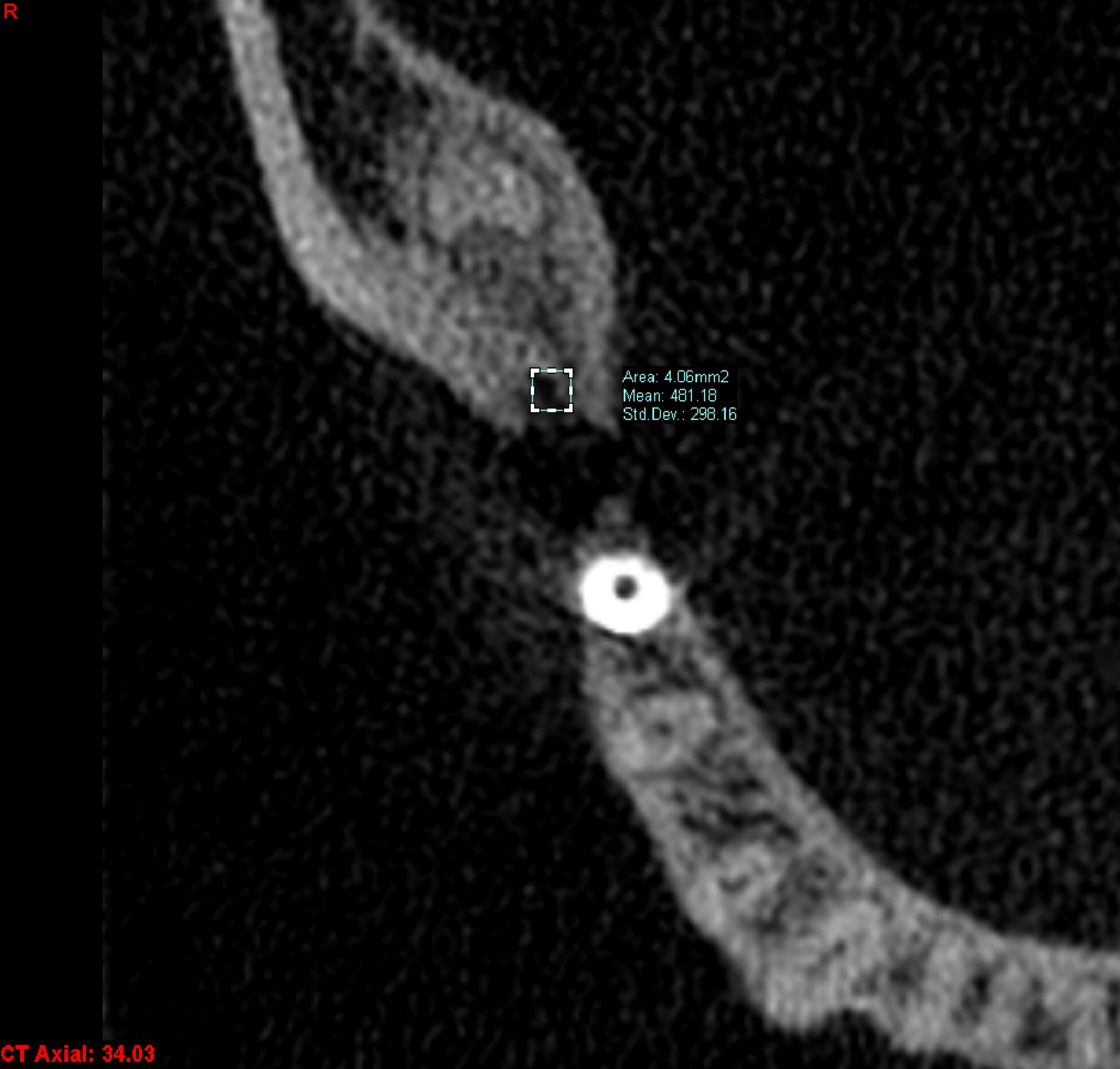
R
Area: 4.06mm2
Mean: 481.18
Std.Dev.: 298.16
CT Axial: 34.03

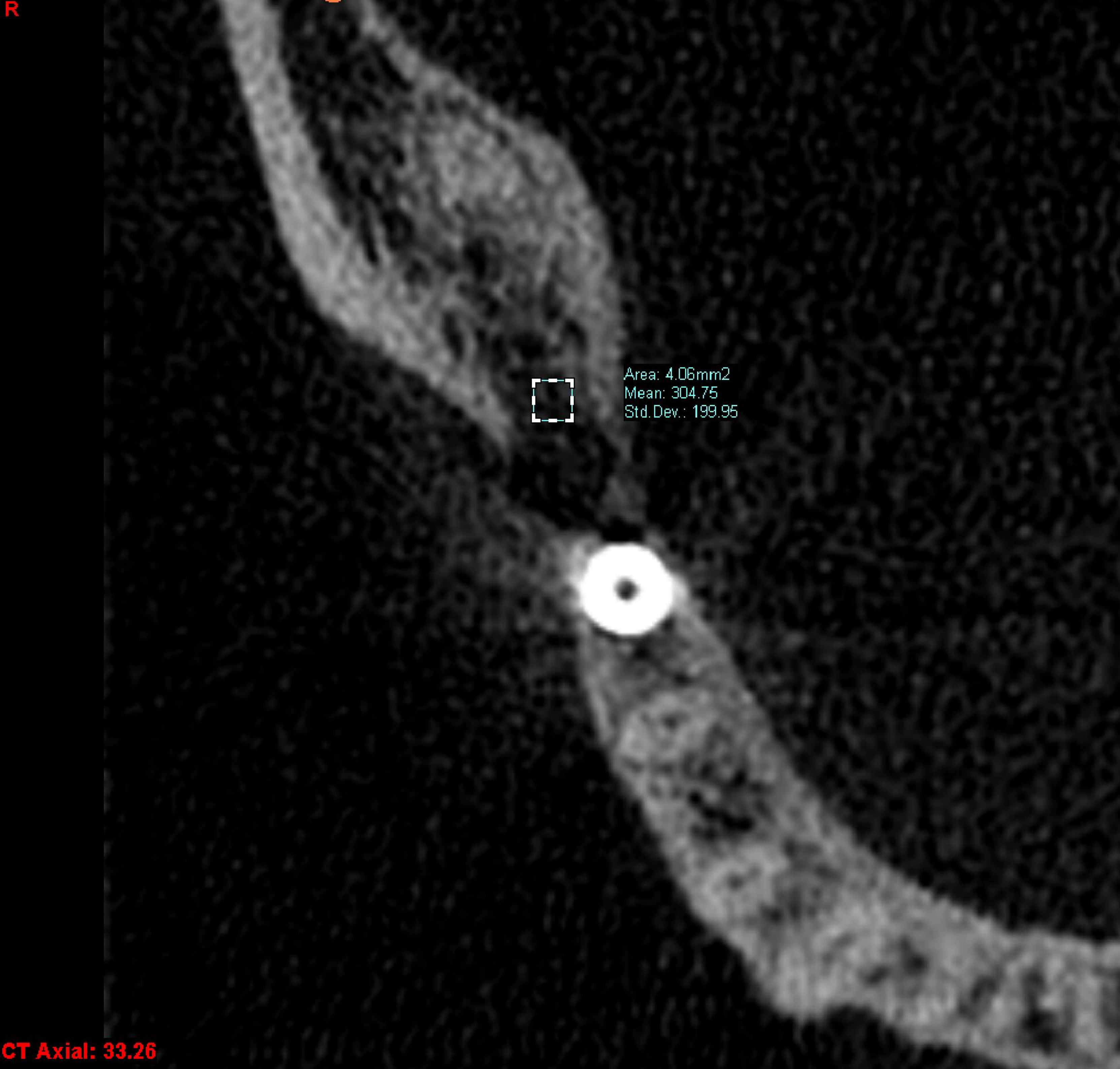
R
Area: 4.06mm2
Mean: 304.75
Std.Dev.: 199.95
CT Axial: 33.26

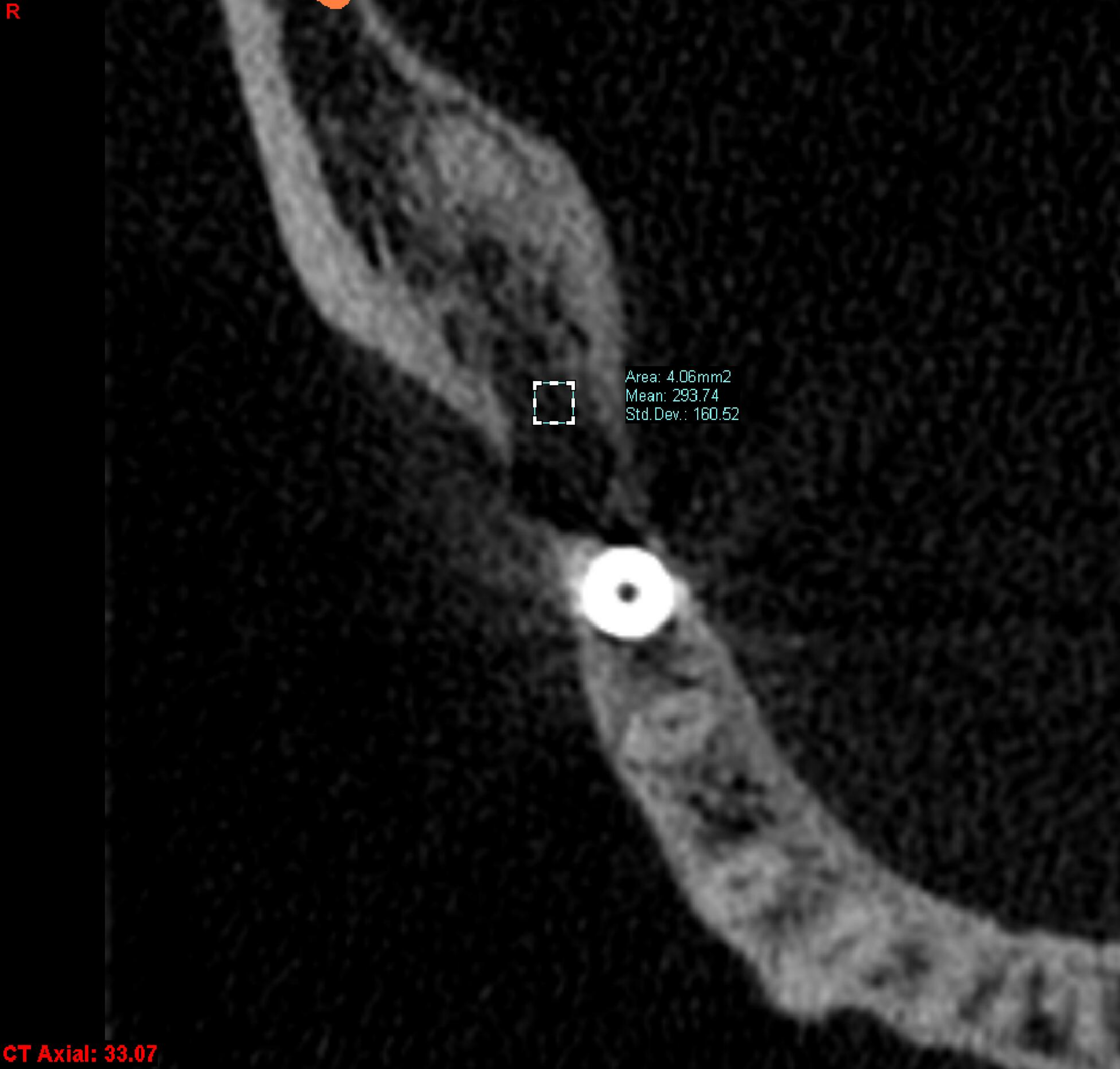
R
Area: 4.06mm2
Mean: 293.74
Std.Dev.: 160.52
CT Axial: 33.07

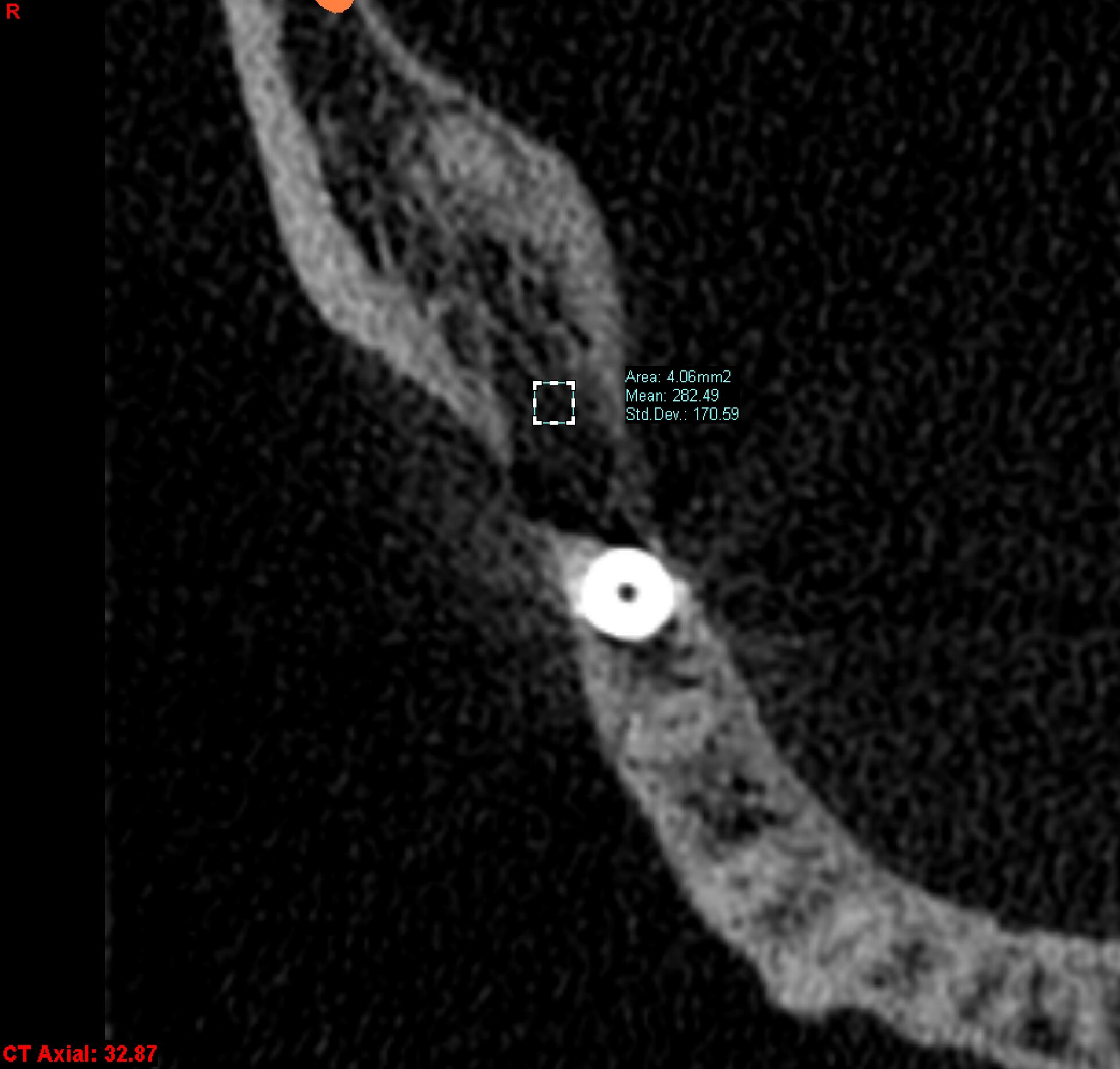
R
Area: 4.06mm2
Mean: 282.49
Std.Dev.: 170.59
CT Axial: 32.87

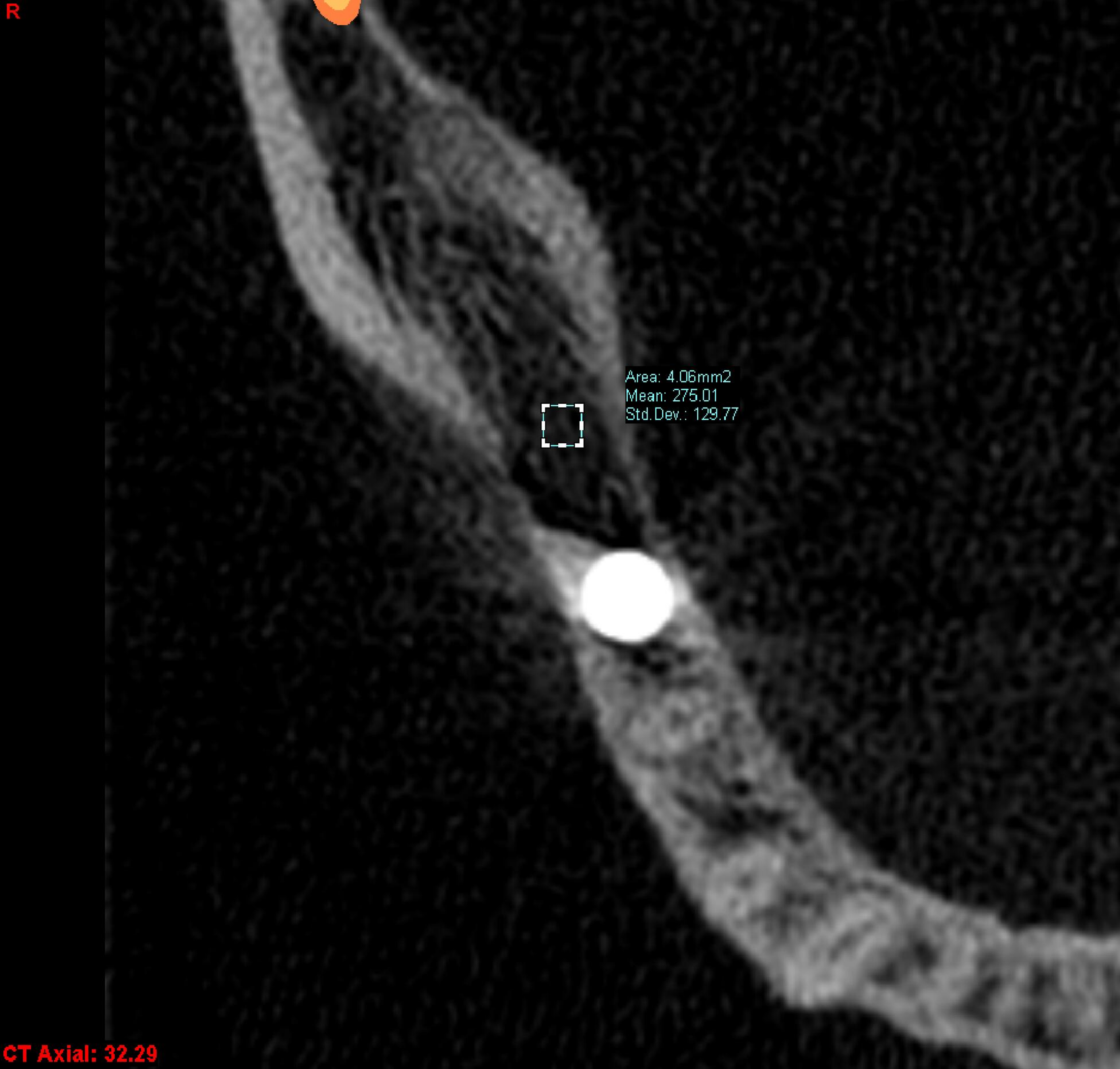
R
Area: 4.06mm2
Mean: 275.01
Std.Dev.: 129.77
CT Axial: 32.29

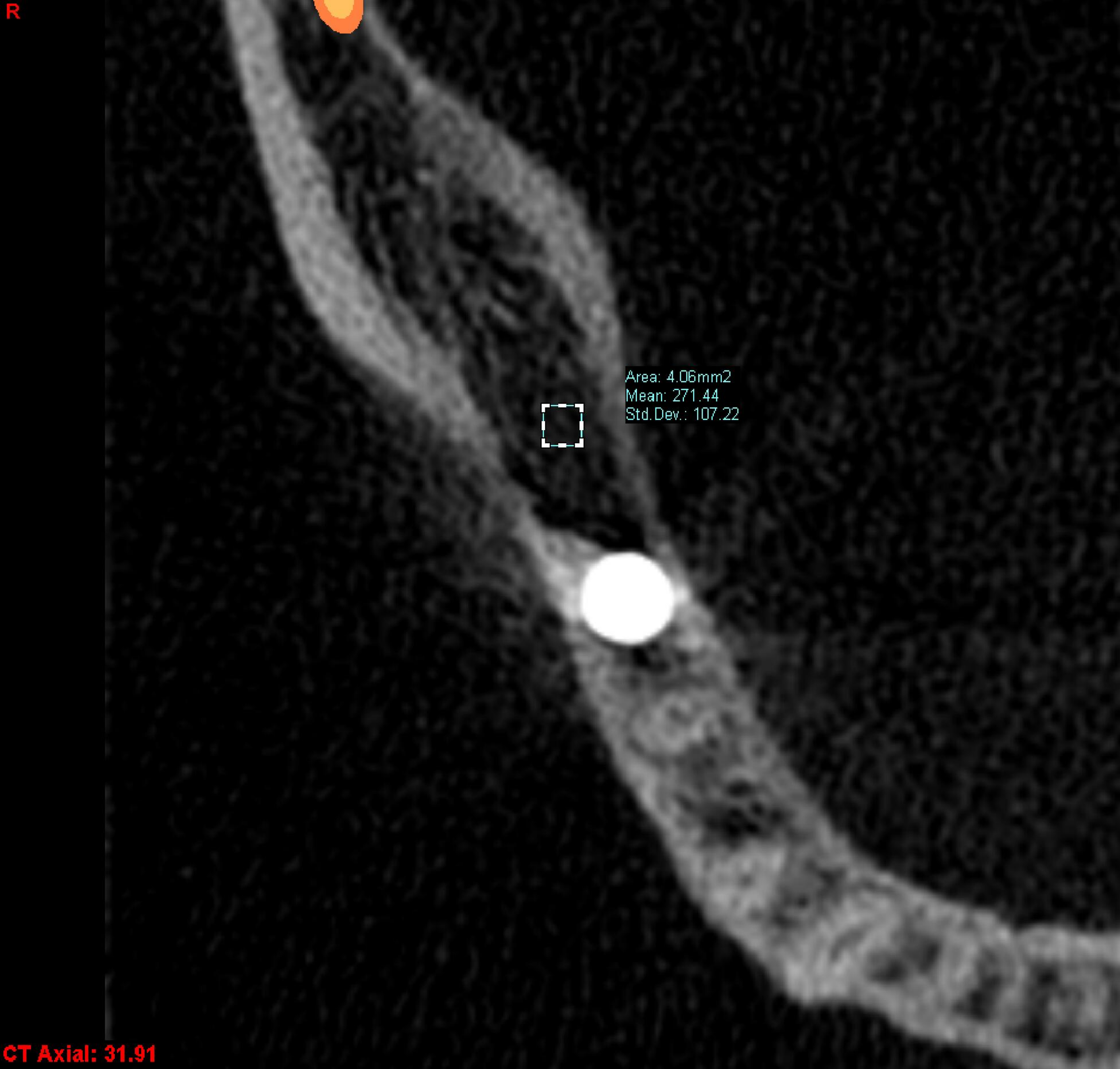
R
Area: 4.06mm2
Mean: 271.44
Std.Dev.: 107.22
CT Axial: 31.91

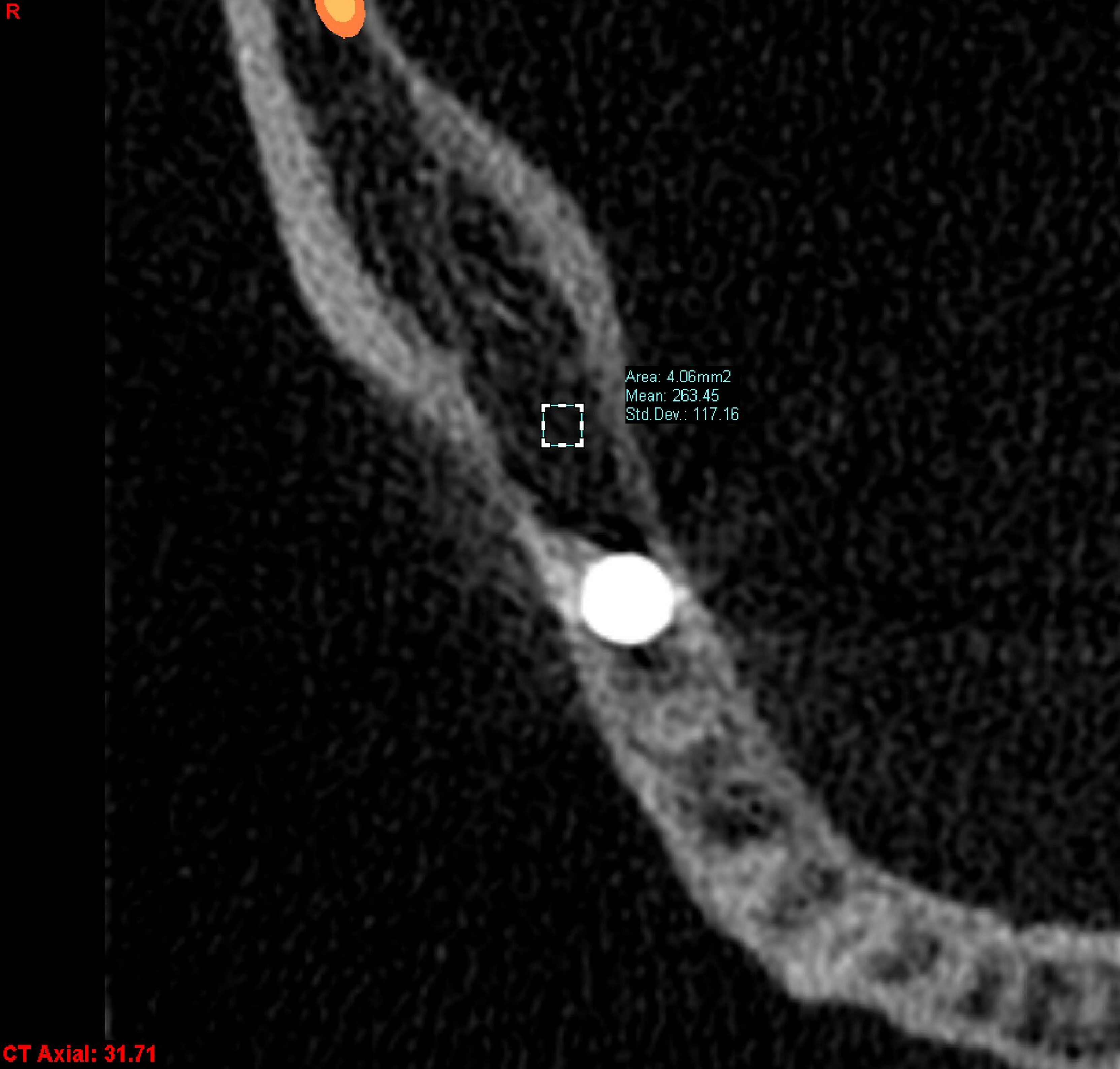
R
Area: 4.06mm2
Mean: 263.45
Std.Dev.: 117.16
CT Axial: 31.71

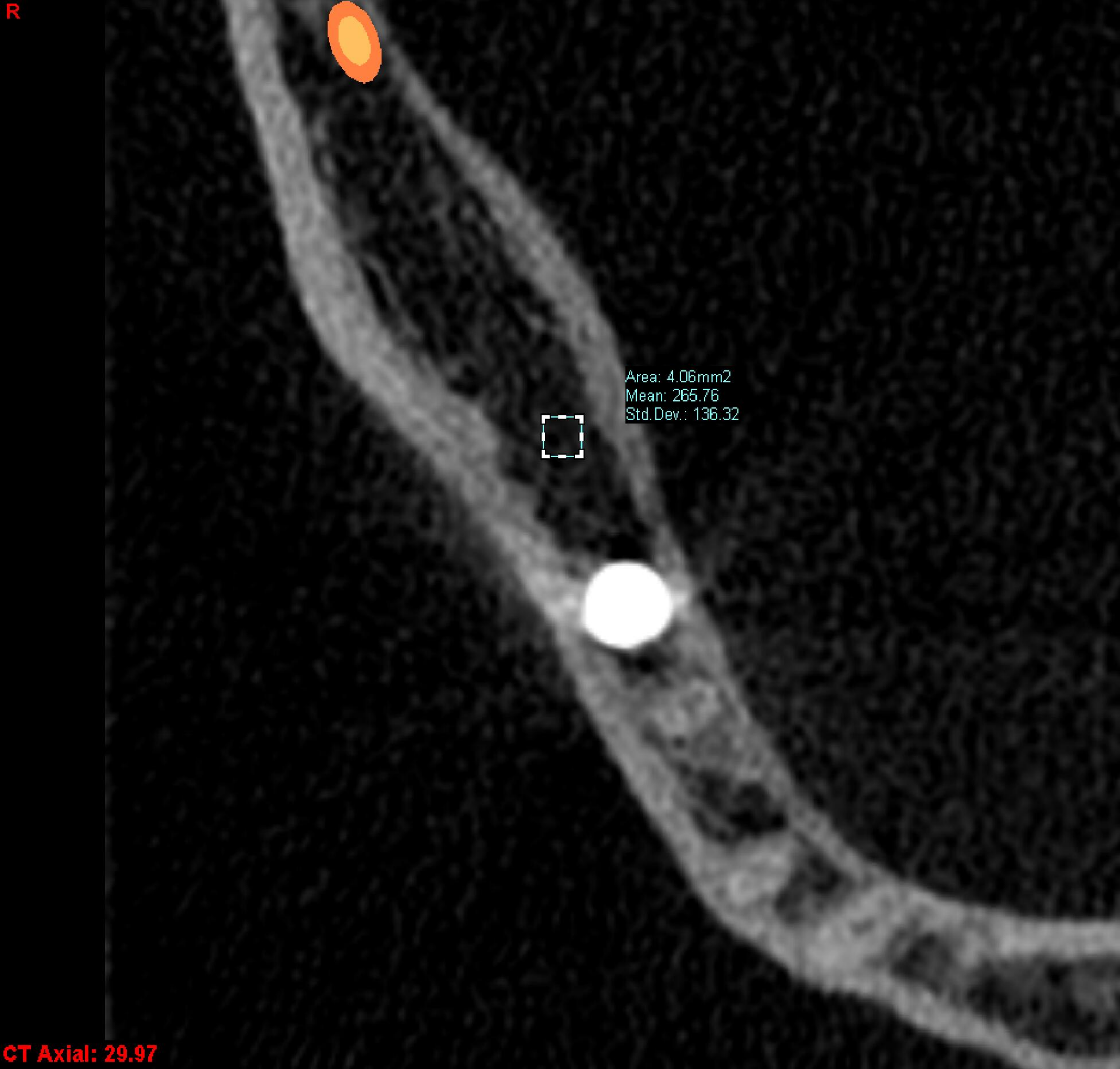
R
Area: 4.06mm2
Mean: 265.76
Std.Dev.: 136.32
CT Axial: 29.97

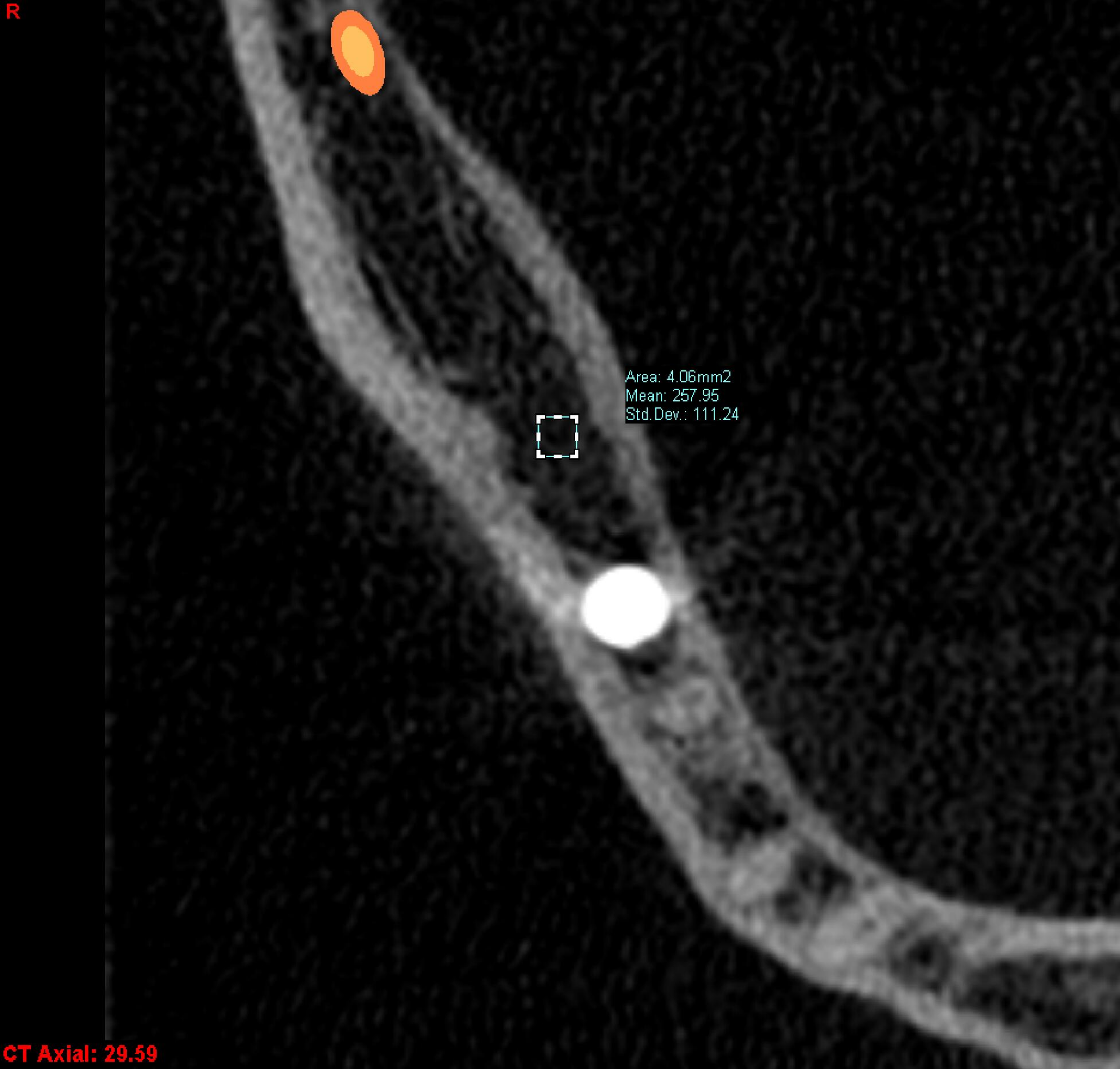
R
Area: 4.06mm2
Mean: 257.95
Std.Dev.: 111.24
CT Axial: 29.59

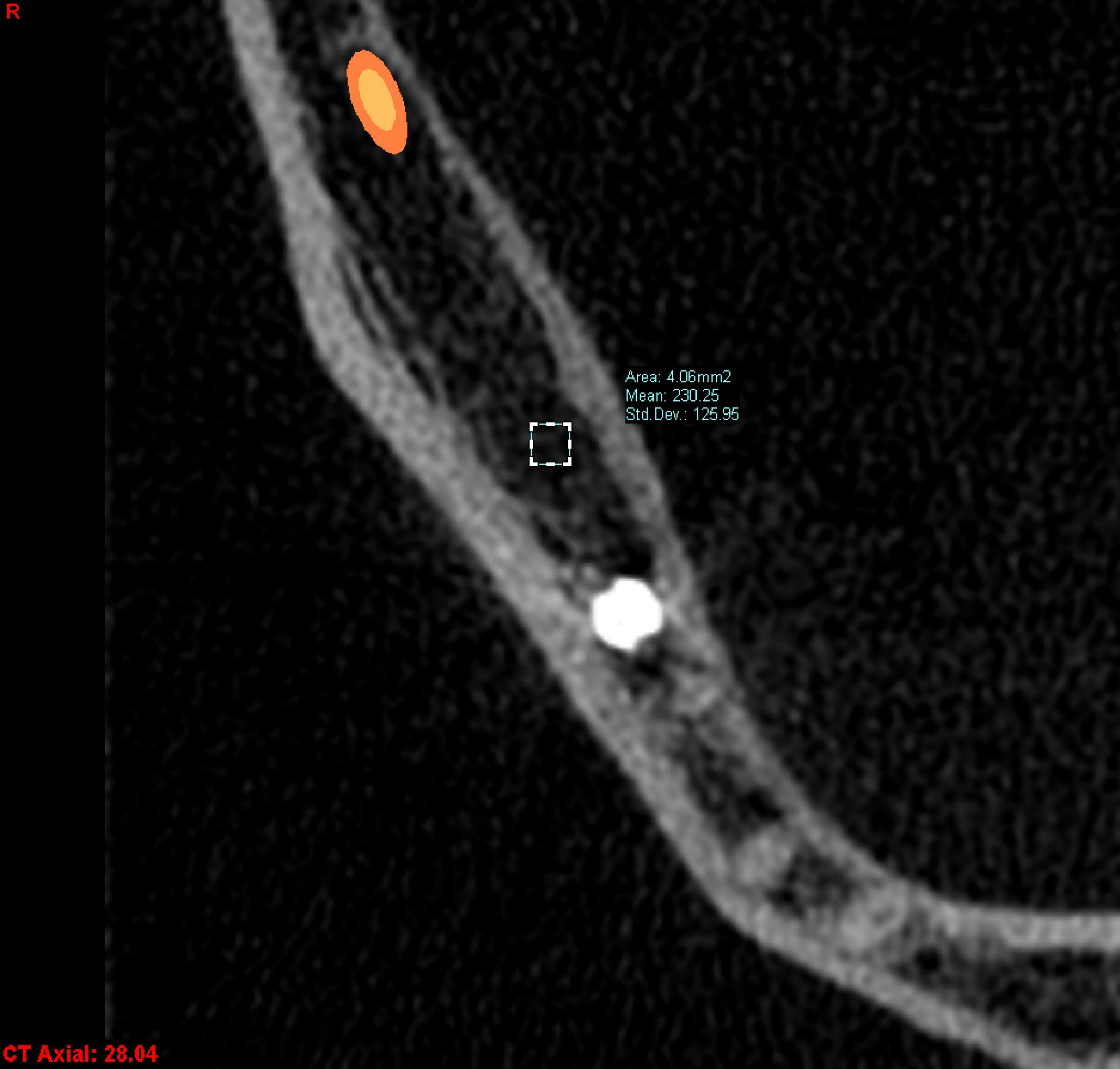
R
Area: 4.06mm2
Mean: 230.25
Std.Dev.: 125.95
CT Axial: 28.04

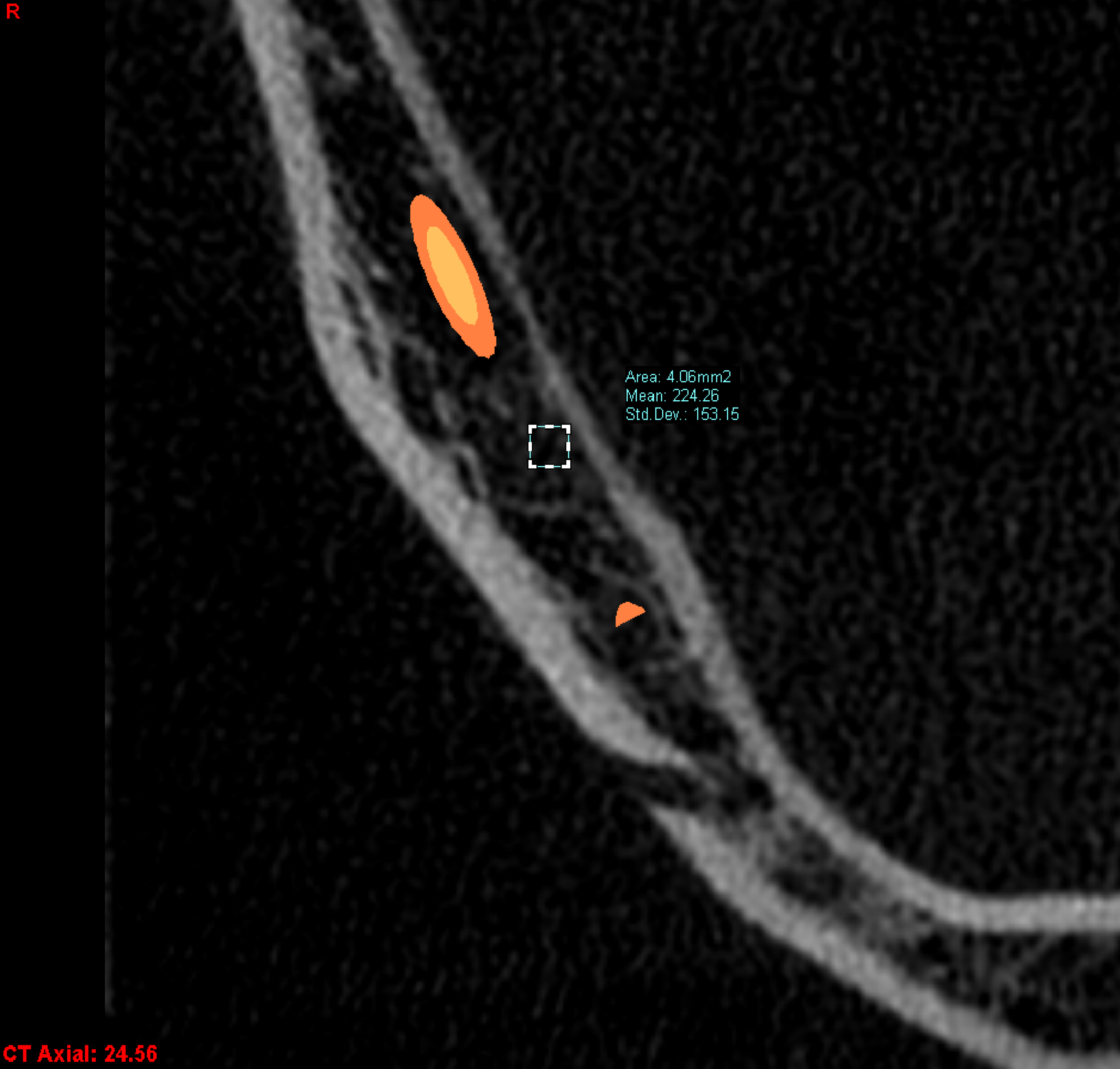
R
Area: 4.06mm2
Mean: 224.26
Std.Dev.: 153.15
CT Axial: 24.56

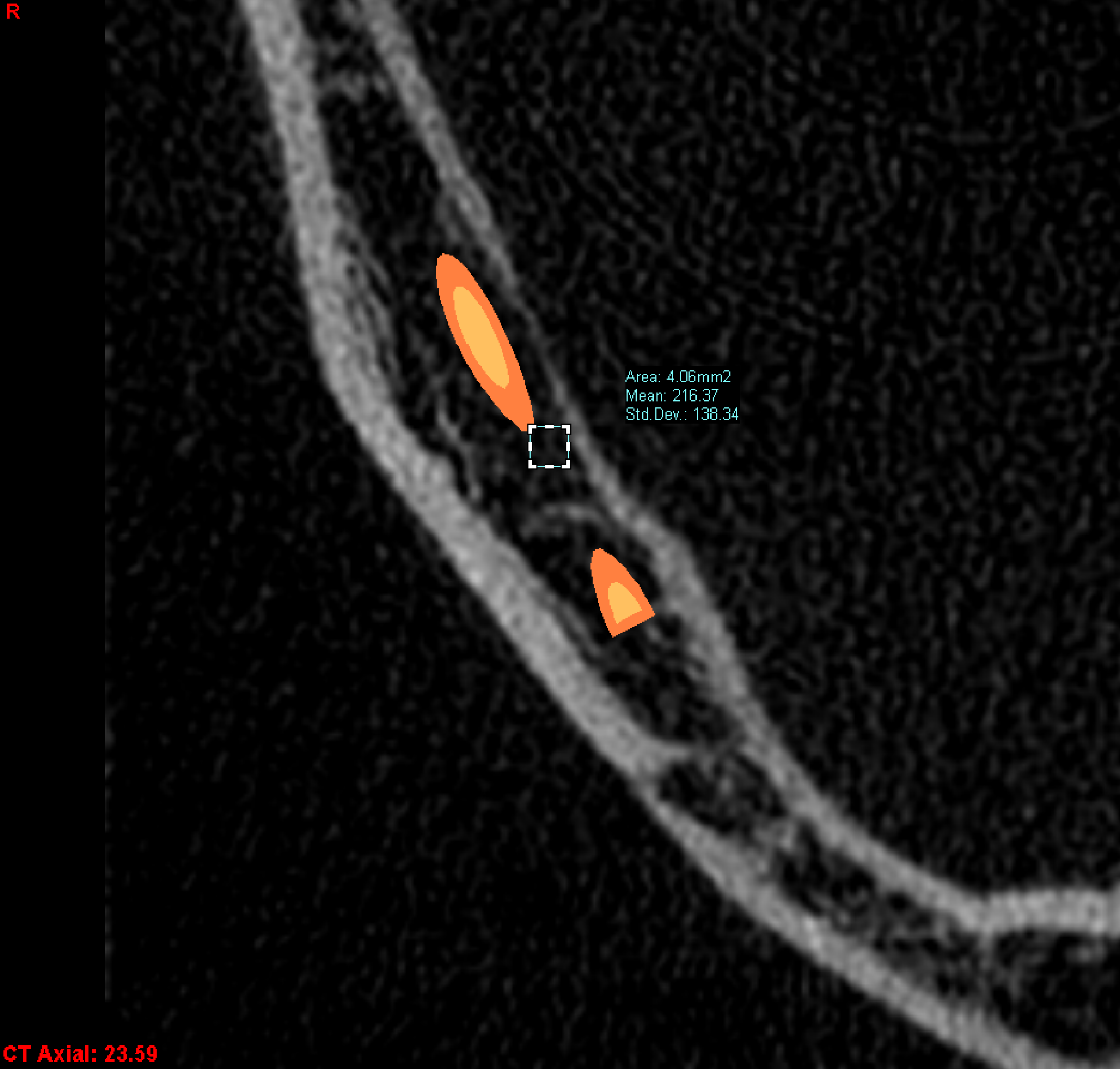
R
Area: 4.06mm2
Mean: 216.37
Std.Dev.: 138.34
CT Axial: 23.59

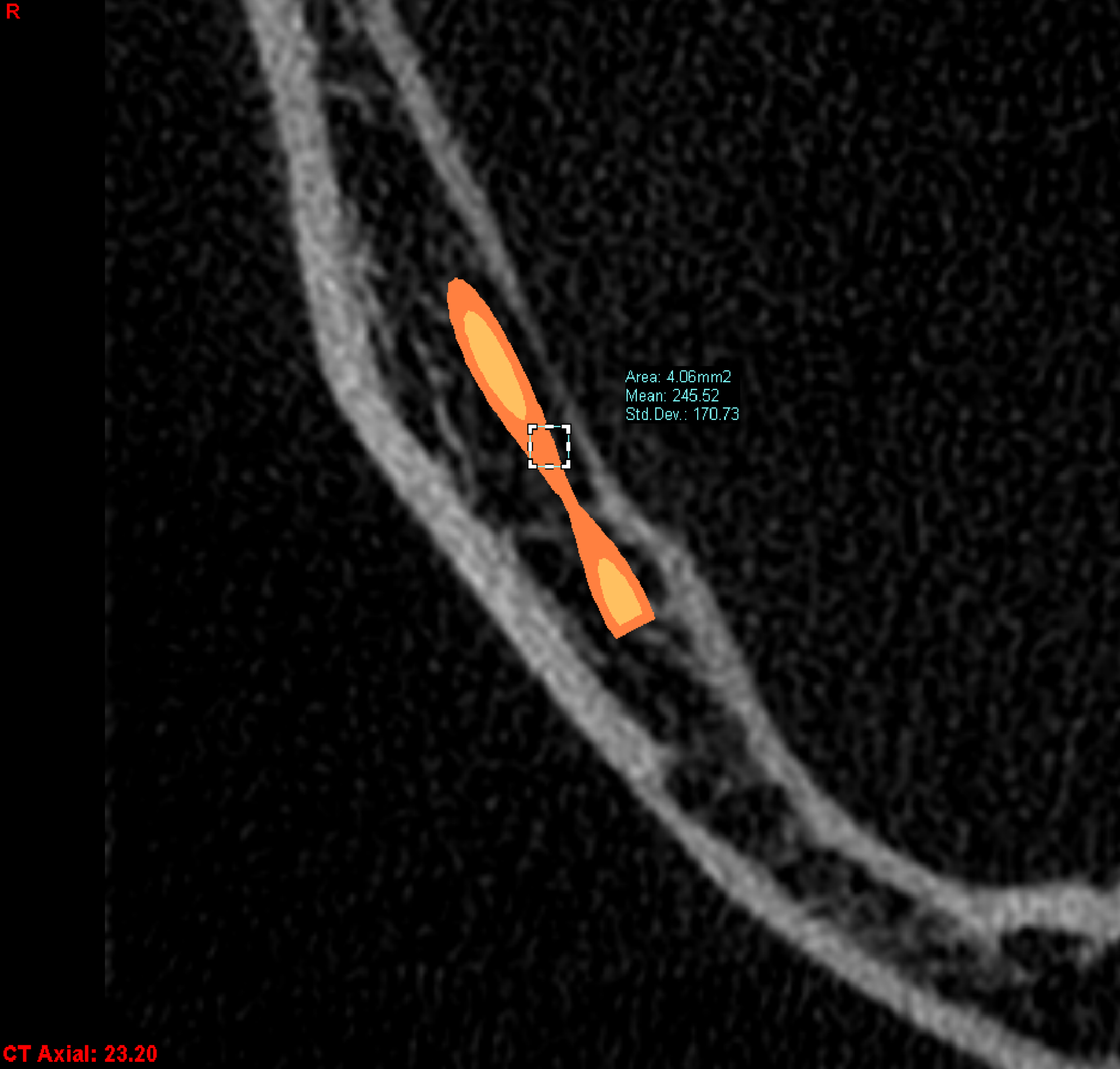
R
Area: 4.06mm2
Mean: 245.52
Std.Dev.: 170.73
CT Axial: 23.20

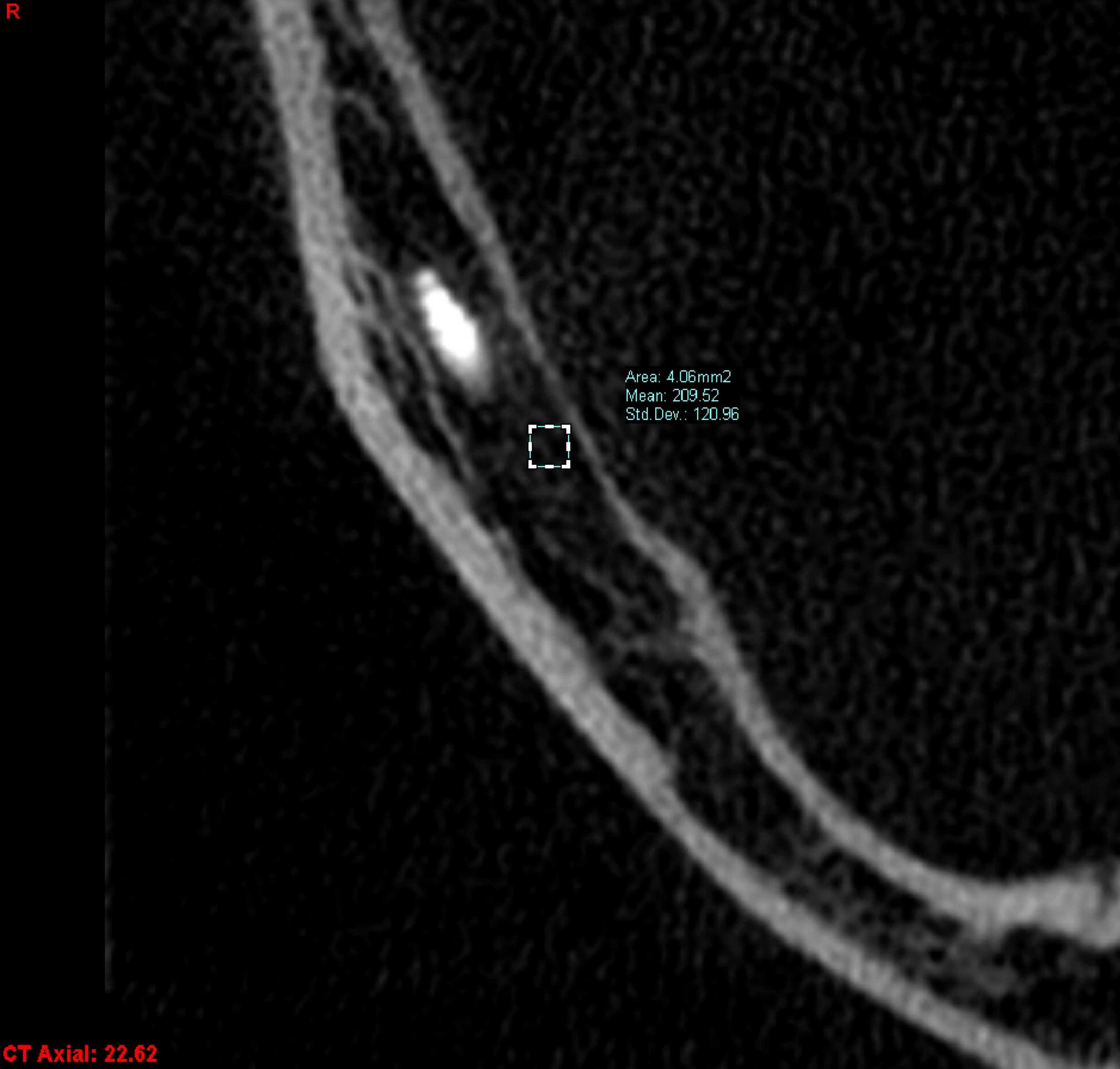
R
Area: 4.06mm2
Mean: 209.52
Std.Dev.: 120.96
CT Axial: 22.62

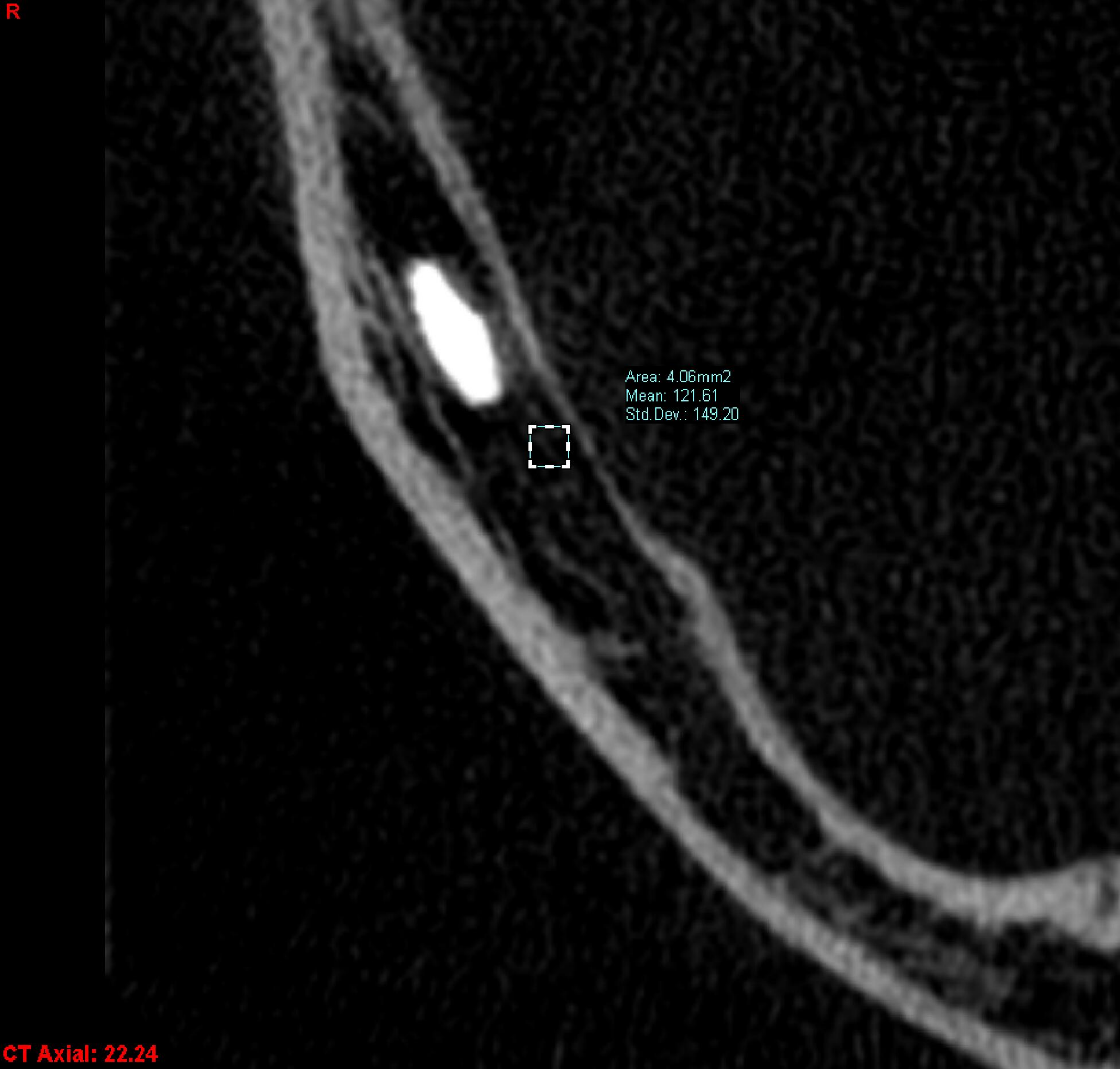
R
Area: 4.06mm2
Mean: 121.61
Std.Dev.: 149.20
CT Axial: 22.24

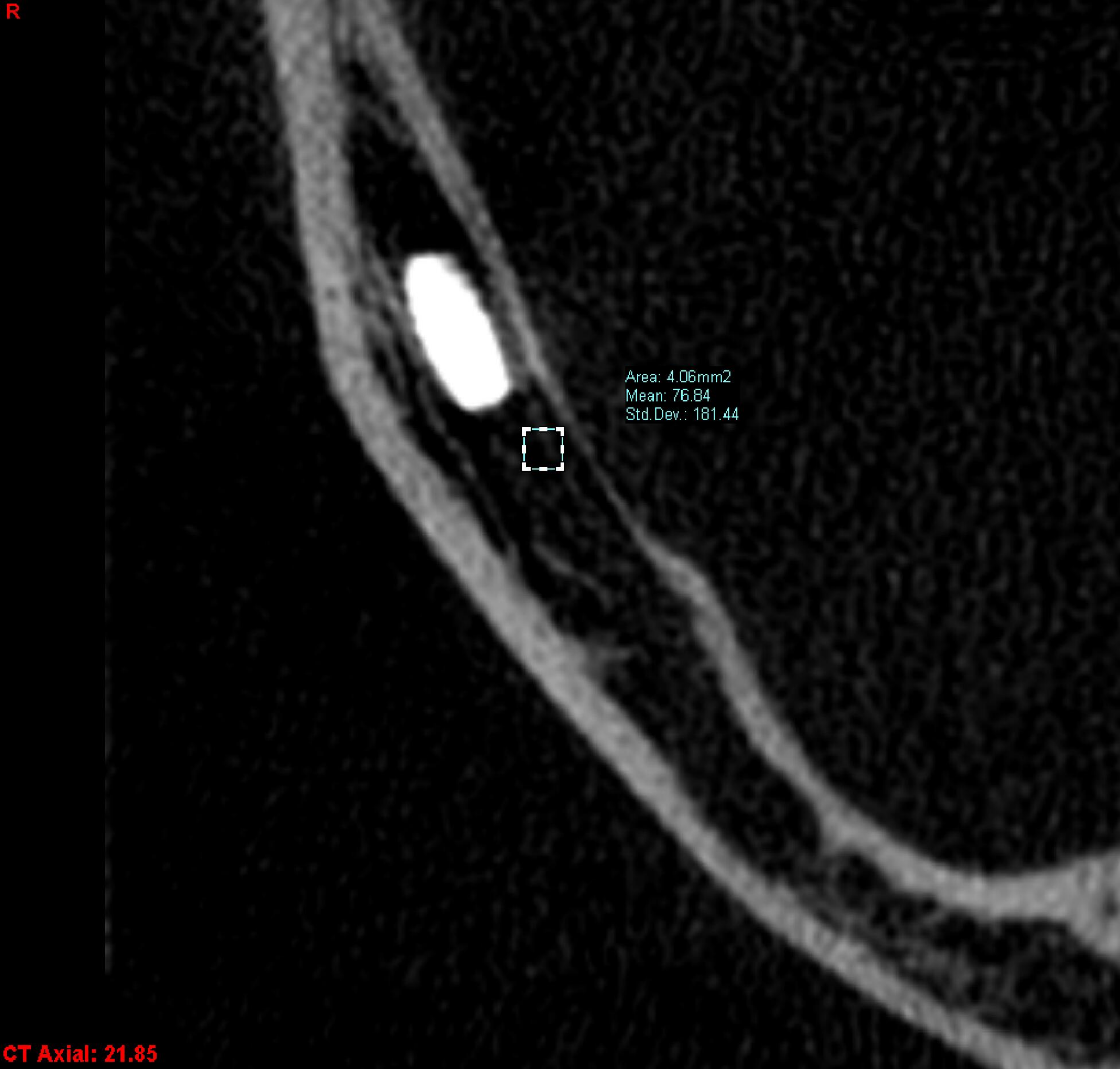
R
Area: 4.06mm2
Mean: 76.84
Std.Dev.: 181.44
CT Axial: 21.85

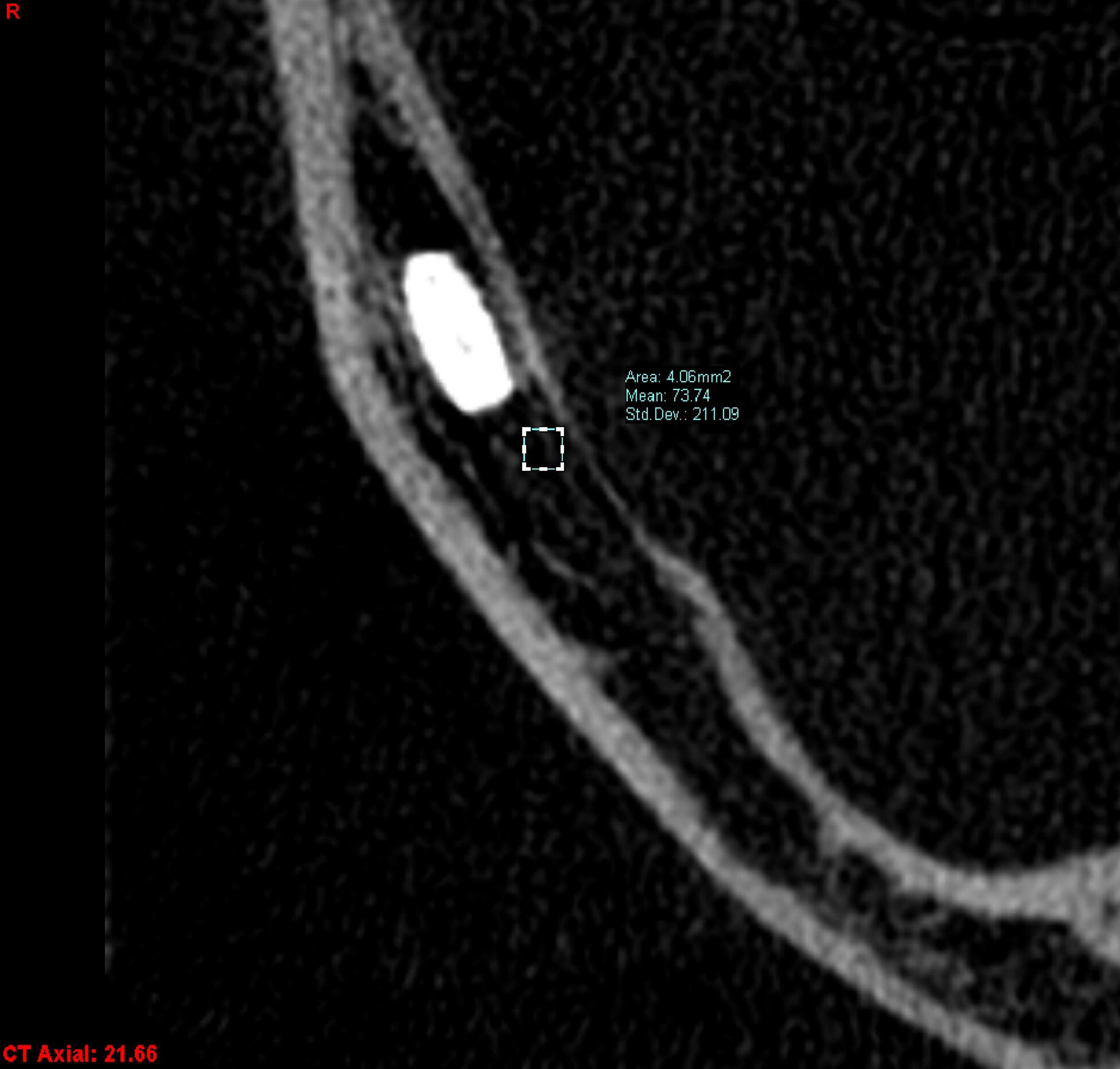
R
Area: 4.06mm2
Mean: 73.74
Std.Dev.: 211.09
CT Axial: 21.66

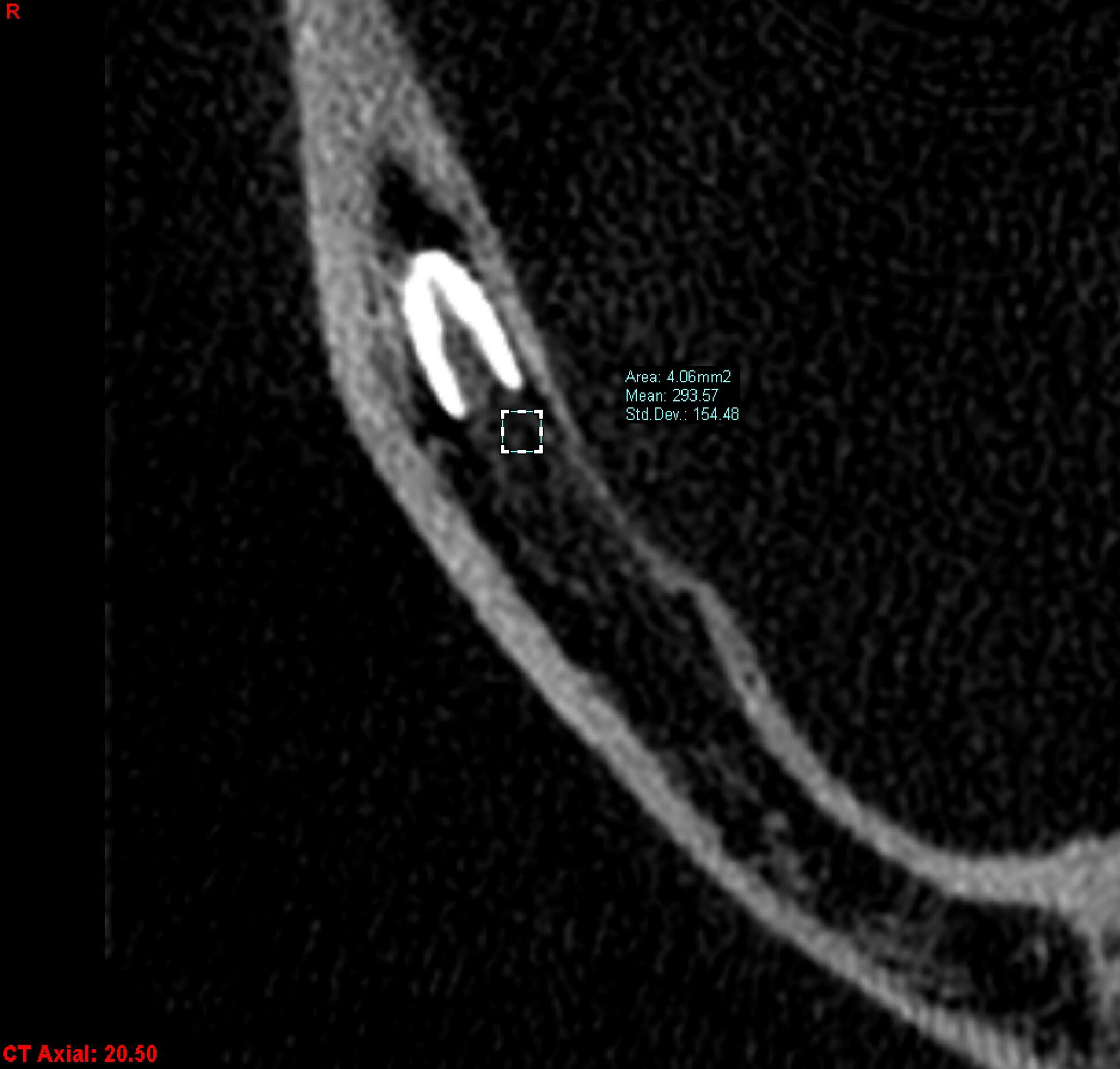
R
Area: 4.06mm2
Mean: 293.57
Std.Dev.: 154.48
CT Axial: 20.50

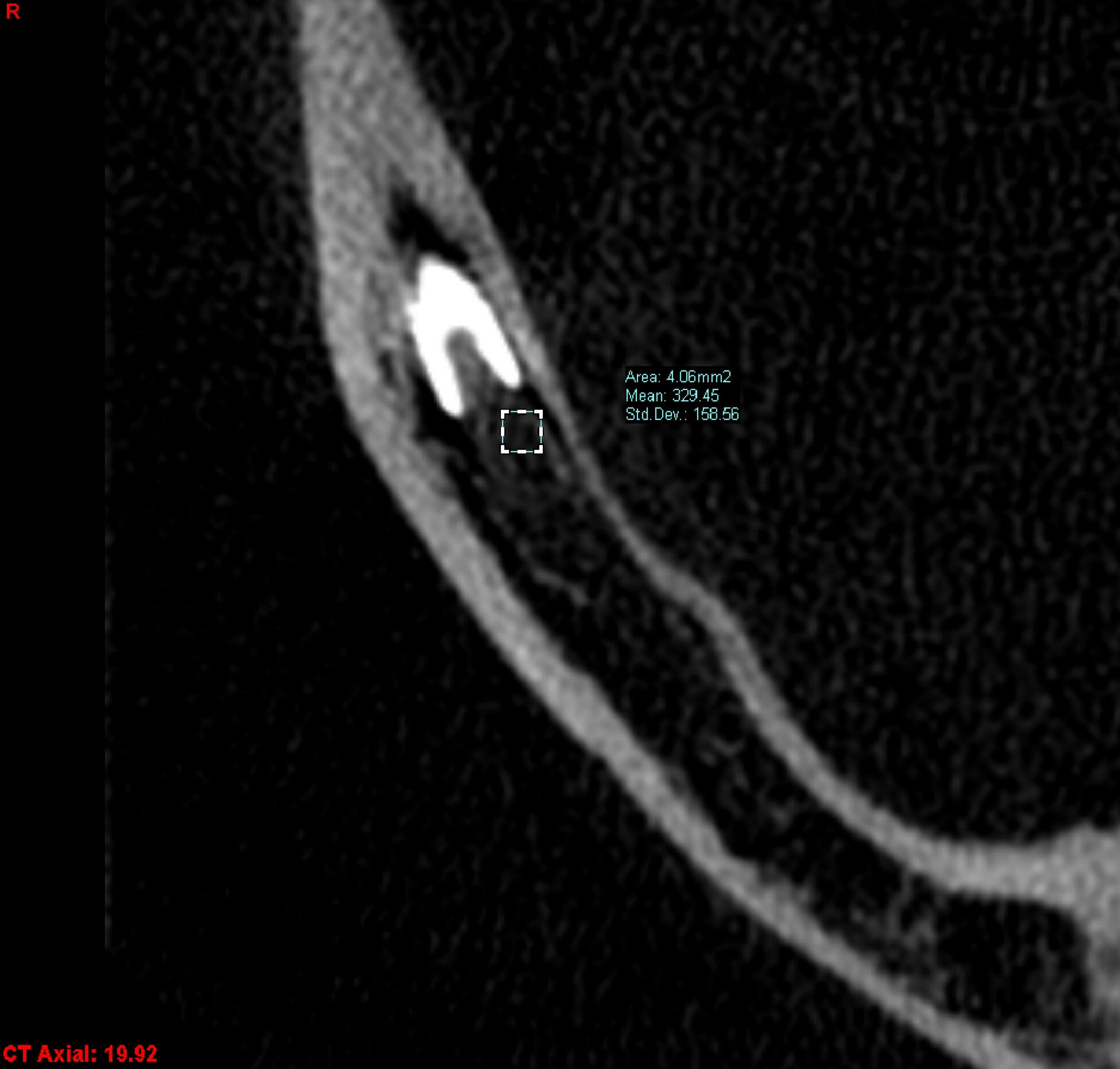
R
Area: 4.06mm2
Mean: 329.45
Std.Dev.: 158.56
CT Axial: 19.92

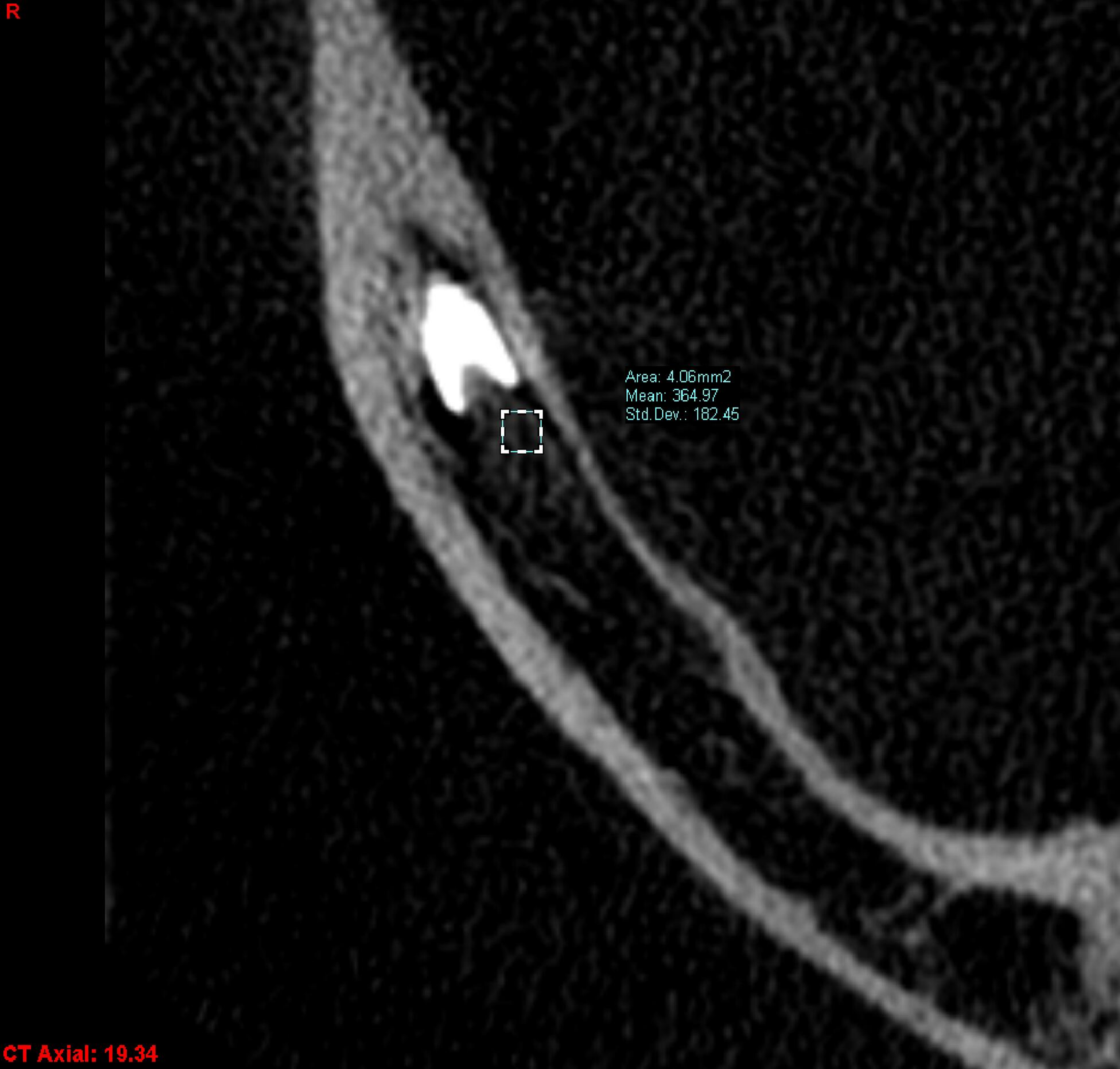
R
Area: 4.06mm2
Mean: 364.97
Std.Dev.: 182.45
CT Axial: 19.34

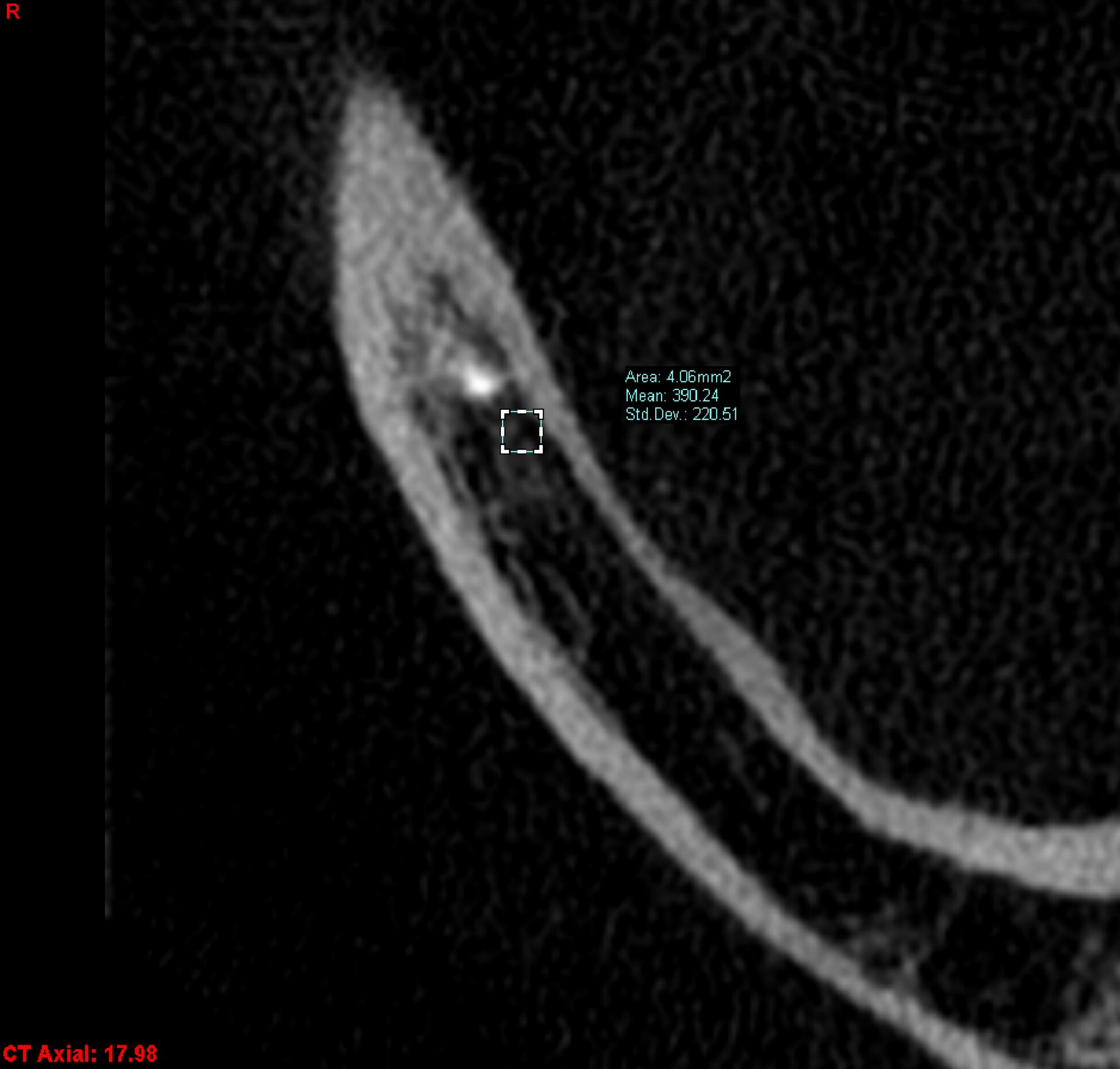
R
Area: 4.06mm2
Mean: 390.24
Std.Dev.: 220.51
CT Axial: 17.98

Bone density of the examined area				
Bone height (mm)	***Area of examination (mm²)***	***Relative Bone Density (HU)***	***St. deviation***	***Notes:***
1	4.06	481.18	298.16	Top of the alveolar ridge
2	4.06	304.75	199.95	
3	4.06	293.74	160.52	
4	4.06	282.49	170.59	
5	4.06	275.01	129.77	
6	4.06	271.44	107.22	
7	4.06	263.45	117.16	
8	4.06	265.76	136.32	
9	4.06	257.95	111.24	
10	4.06	230.25	125.95	
11	4.06	224.26	153.15	
12	4.06	216.37	138.34	
13	4.06	245.52	170.73	Upper border of the mandibular canal
14	4.06	121.61	149.2	
15	4.06	73.74	211.09	
16	4.06	293.57	151.48	Lower border of the mandibular canal
